# Antimicrobial resistance in urinary pathogens among Swedish nursing home residents remains low: a cross-sectional study comparing antimicrobial resistance from 2003 to 2012

**DOI:** 10.1186/1471-2318-14-30

**Published:** 2014-03-13

**Authors:** Pär-Daniel Sundvall, Marie Elm, Ronny Gunnarsson, Sigvard Mölstad, Nils Rodhe, Lars Jonsson, Peter Ulleryd

**Affiliations:** 1 Research and Development Unit, Primary Health Care in Southern Älvsborg County, Sven Eriksonsplatsen 4, SE-503 38, Borås, Sweden; 2 Department of Public Health and Community Medicine, Institute of Medicine, the Sahlgrenska Academy, University of Gothenburg, Box 100, SE-405 30 Gothenburg, Sweden; 3 Sandared Primary Health Care Centre, Sandared, Sweden; 4 Health Care Unit, Borås Municipality, Våglängdsgatan 21 B, SE-507 41 Borås, Sweden; 5 Cairns Clinical School, School of Medicine and Dentistry, James Cook University, Cairns Base Hospital, PO Box 902, Cairns, QLD 4870, Australia; 6 Department of Clinical Sciences, General Practice, Lund University, CRC, Hus 28, Plan 11, Jan Waldenströms gata 35, Malmö, SE-205 02, Sweden; 7 Centre for Clinical Research, Dalarna, Sweden and Falu Vårdcentral, Södra Mariegatan 18, SE-791 70 Falun, Sweden; 8 Department of Public Health and Caring Sciences, Family Medicine, Uppsala University, Uppsala, Sweden; 9 Bio Imaging and Laboratory Medicine Unit, Södra Älvsborg Hospital, SE-501 82 Borås, SE-501 82, Sweden; 10 Department of Infectious Diseases, Institute of Biomedicine, the Sahlgrenska Academy, University of Gothenburg, Sweden and Department of Communicable Disease Control, Västra Götalandsregionen, SE-501 82 Borås, Sweden

**Keywords:** Drug resistance, Bacterial, Bacteriuria, Nursing homes, Homes for the aged, Urinary tract infections, Antibiotics

## Abstract

**Background:**

There are several risk factors for the colonisation, infection and spreading of antibiotic resistant bacteria among elderly residents of nursing homes. An updated estimate of the native prevalence of antimicrobial resistance in uropathogens among Swedish nursing home residents is needed.

**Methods:**

Urine specimens were collected for culture and antimicrobial susceptibility testing against mecillinam, ampicillin, cefadroxil, trimethoprim, nitrofurantoin and quinolones from the residents of 32 and 22 nursing homes, respectively. The residents were capable of providing a voided urine sample in 2003 and 2012. In 2012 urine specimens were also collected from residents with urinary catheters. Any antibiotic treatment during the previous month was registered in 2003 as well as hospitalisation and any antibiotic treatment during the previous six months in 2012.

**Results:**

The proportion of positive urine cultures was 32% (207/651) in voided urine specimens in 2003, 35% (147/421) in 2012, and 46% (27/59) in urine samples from catheters in 2012. *Escherichia coli* (*E. coli*) was the most commonly occurring bacteria.

Resistance rates in *E. coli* (voided urine specimens) in 2012 were; ampicillin 21%, trimethoprim 12%, mecillinam 7.7%, ciprofloxacin 3.4%, cefadroxil 2.6% and nitrofurantoin 0.85%. There were no significant changes in the average resistance rates in *E. coli* for antibiotics tested 2003–2012.

In 2012, two isolates of *E. coli* produced extended spectrum beta-lactamase enzymes (ESBL) and one with plasmid mediated AmpC production.

Any antibiotic treatment during the previous month increased the risk for resistance in *E. coli*, adjusted for age and gender; for mecillinam with an odds ratio (OR) of 7.1 (2.4-21; p = 0.00049), ampicillin OR 5.2 (2.4-11; p = 0.000036), nalidixic acid OR 4.6 (1.4-16; p = 0.014) and trimethoprim OR 3.9 (1.6-9.2; p = 0.0023). Hospitalisation during the previous six months increased the risk for antibiotic resistance in *E. coli* to ampicillin, ciprofloxacin and any antimicrobial tested, adjusted for age, gender and antibiotic treatments during the previous six months.

**Conclusions:**

The average rates of antimicrobial resistance were low and did not increase between 2003 and 2012 in *E. coli* urinary isolates among Swedish nursing home residents. Antibiotic treatment during the previous month and hospitalisation during the previous six months predicted higher resistance rates.

## Background

Antimicrobial resistance is on the rise and a cause of major concern in many countries [1]. Extensive antibiotic prescription is related to a higher prevalence of antibiotic resistant bacteria [2-7]. The elderly are prescribed antibiotics more frequently than younger adults, and antibiotic courses are common at nursing homes [8-14]. There are several risk factors for colonisation, infection and spreading of antibiotic resistant bacteria among elderly residents of nursing homes such as catheters, decubitus ulcers and other wounds [15,16].

There are some studies of antimicrobial resistance in uropathogens among elderly residents at nursing homes, however, there are considerable differences in resistance rates between countries [17-22].

Antimicrobial resistance in urinary pathogens is still favourable in Sweden within an international perspective [23]. There was a low prevalence of ESBL-producing bacteria in faecal samples collected in Swedish nursing homes in 2008 [24]. However, between 2008 and 2010 ESBL faecal carriage increased both in the community and at a university hospital in Sweden [25].

Resistance was generally low in 183 screening urine samples from Swedish residents of nursing homes in 2008–2010 [26]. There was a tendency towards higher antimicrobial resistance among strains isolated in 2010 from nursing home residents with indwelling bladder catheters compared to all urine strains at that laboratory [27].

It is important to frequently update information concerning the native prevalence of antimicrobial resistance in uropathogens among residents of nursing homes, and be on the alert for significant changes. Any changes might affect empirical treatment of urinary tract infections (UTI) and antibiotic stewardship in nursing homes.

The primary aim of this study was to describe antimicrobial resistance rates in uropathogens among residents of Swedish nursing homes in 2012 and compare these to the rates from 2003.

The second aim of the study was to determine if antibiotic treatment within the previous month or hospitalisation within the previous six months predicted higher resistance rates in uropathogens among residents of nursing homes.

## Methods

From January to March of 2003 and 2012, a single voided urine specimen was collected from all included residents of the participating nursing homes for the elderly. In 2012, a single urine specimen was also collected from residents with indwelling urinary catheters. In 2003, the 32 participating nursing homes were located in four municipalities in southwestern Sweden. Two of these municipalities had 22 participating nursing homes in 2012. The attending nurses were provided detailed verbal and written information for the procedure.

The data from 2003 was collected as part of another study evaluating dipstick urinalysis among elderly residents of nursing homes [28] and evaluating the relationship between nonspecific symptoms and bacteriuria [29]. Both parts of this study, the data gatherings in 2003 and 2012, were approved by the Regional ethical review board of Gothenburg University (D-nr Ö 410–02 and 578–11).

### Inclusion and exclusion criteria

Residents of the participating nursing homes, regardless of UTI symptoms were requested to participate. Those accepting participation were included after having met the following inclusion criteria:

• Permanent residence in nursing homes for the elderly (regardless of gender)

• Presence at the nursing home during the study

• Participation approval

• No indwelling urinary catheter in 2003, only voided urine specimens were collected

• Both voided urine specimens and specimens from indwelling urinary catheters were collected in 2012

• Sufficiently continent to leave a voided urinary specimen (unless the resident had an indwelling urinary catheter in 2012)

• Residents with dementia included if cooperative when collecting urine samples

• No urostomy

• No regularly clean intermittent catheterisation

• No ongoing dialysis

• Not terminally ill

The following exclusion criterion was used:

• If the resident did not agree to participate or wished to discontinue participation

### Statement of consent

Residents were informed of the study verbally and in writing. Informed approval for participation in the study was collected from decision-capable individuals choosing to participate in the study. However, a considerable number of participants consisted of residents with varying degrees of dementia. If incapable of understanding the information and thereby possessing a reduced decision capability, these residents only participated so long as they did not oppose participation, and under the condition that appointed representative or relatives did not oppose participation after having taken part of the study information.

### Study protocol

In addition to collecting the urine sample, the attending nurse made an entry in the study protocol, once for each included resident, whether having ongoing or previous antibiotic treatment within the last month or diabetes mellitus. In 2012, the attending nurse also registered overnight admissions to hospital, any antibiotic treatment during the last six months, and an eventual diagnosis of dementia.

### Laboratory tests

The personnel at the nursing homes were instructed to collect a mid-stream morning sample, or a voided urine specimen with so long a bladder incubation time as possible. Dipstick urinalysis was carried out at the nursing home. The microbiology laboratory was provided information on the outcome of the dipstick urinalysis as well as information on any UTI symptoms from the attending nurse. The urine specimens were cultured at the microbiology laboratory in Borås according to clinical routine procedure. The urine samples were chilled before transport and usually arrived at the laboratory within 24 hours.

The laboratory fractionated 10 μl urine on a cystine-lactose-electrolyte deficient agar (CLED) and a Columbia blood agar. Plates were incubated overnight (minimum 15 h) at 35–37°C. CLED plates were incubated in air and Columbia plates were incubated in 5% CO_2_. The latter was further incubated for 24 hours if no growth occurred after the first incubation. Growth of bacteria was considered significant if the number of colony-forming units (CFU)/mL was ≥10^5^. However, at signs of possible UTI such as a positive nitrite dipstick, leukocyte esterase dipstick >1, fever, frequency, urgency or dysuria, the cut-off point was ≥10^3^ for patients with a growth of *Escherichia coli (E. coli)* and male patients with *Klebsiella* species (spp) and *Enterococcus faecalis (E. faecalis).* For symptomatic women harbouring the two latter species the cut-off level was set at ≥10^4^. At these lower cut-off points and with no symptoms or signs of possible UTI, the urine cultures were classified as sparse growth. For the purpose of this study, both significant and sparse growth were defined as a positive urine culture. In case of several detected species, *E. coli* was selected in favour of secondary pathogens such as *Klebsiella* spp. and in case of several *E. coli,* the most prevalent isolate was selected. Growth of mixed flora was classified as a negative urine culture.

The antimicrobial susceptibility of bacteria was determined according to the disk diffusion method described by the Swedish Reference Group for Antibiotics (SRGA) at the time. In 2012 antimicrobial susceptibility tests followed guidelines and breakpoints proposed by the European Committee on Antimicrobial Susceptibility Testing (EUCAST) for the standardised disk diffusion test [30]. In 2003 nalidixic acid was used as a screening disk for any quinolone resistance; in 2012 isolates were only tested for ciprofloxacin resistance according to national guidelines. Bacterial isolates with suspected extended spectrum beta-lactamase (ESBL) production were confirmed as ESBL-producing bacteria by the reference laboratory at the Swedish Institute for Communicable Disease Control.

### Statistical analysis

The population was described by number of individuals, age and gender in 2003 and 2012. Any differences between 2003 and 2012 were compared either by Pearson chi-square or *T*-test.

Differences between bacterial species and resistance patterns between 2003 and 2012 were analysed by Pearson chi-square, and when appropriate, Fisher’s exact test.

To evaluate the impact of previous antibiotic treatments during the last month on antimicrobial resistance rates, adjusted for age and gender, logistic regressions were performed for those with *E. coli* in voided urine specimens collected in 2003 and 2012. The outcome of antimicrobial susceptibility testing was used as the dependent variable, and any antibiotic treatment during the previous month, age and gender as the independent variables. One regression was made for each antibiotic commonly used to treat UTI.

To evaluate the impact of hospitalisation during the previous six months on antimicrobial resistance rates adjusted for age, gender and any antibiotic treatment during the previous six months, logistic regressions were performed for those with *E. coli* in voided urine specimens collected in 2012. The outcome of antimicrobial susceptibility testing was used as the dependent variable, and any hospitalisation and any antibiotic treatment during the previous six months, as well as age and gender, as independent variables. One regression was made for each antibiotic commonly used to treat UTI and finally for any antimicrobial resistance tested. Cramer’s V was calculated to evaluate any correlation between any hospitalisation and any antibiotic treatment during the previous six months.

IBM SPSS Statistics version 21 was used for statistical analysis.

## Results

### Studied population in 2003

**Figure 1:**
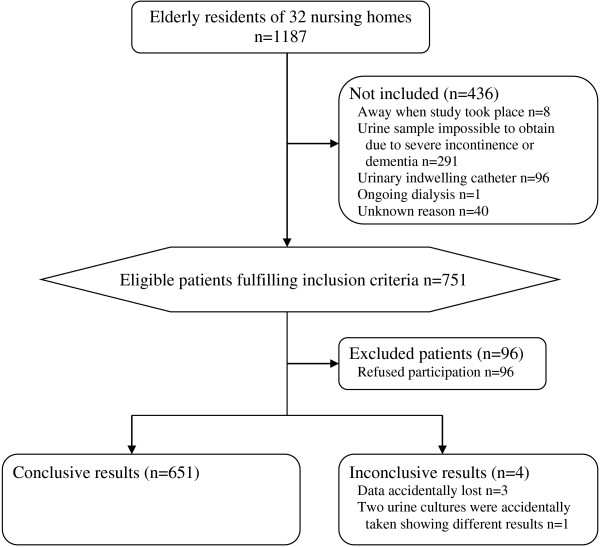
Participant flow chart 2003.

In 2003, 751 of 1187 individuals in 32 nursing homes fulfilled the inclusion criteria, and 655 (87%) accepted participation (Figure 1). Voided urine samples were provided from 651 individuals, 482 (74%) women and 169 (26%) men. Women’s ages (mean 86 years, SD 7.4, range 46–102) were slightly higher than men’s (mean 82 years, SD 7.8, range 54–99) (p < 10^-6^). 100/651 (15%) suffered from diabetes mellitus. When the urine specimens were collected, 26/651 (4.0%) were undergoing antibiotic treatment. Another 61/651 (9.4%) had no ongoing antibiotic treatment when the urine specimens were collected, but had received antibiotics during the previous month. The antibiotic treatment history was, however, unknown for 12/651 (1.8%). The presence of new or increased urinary symptoms occurring during the previous week was; for dysuria 5/651 (0.77%) and urinary urgency 6/651 (0.92%).

### Studied population in 2012

**Figure 2:**
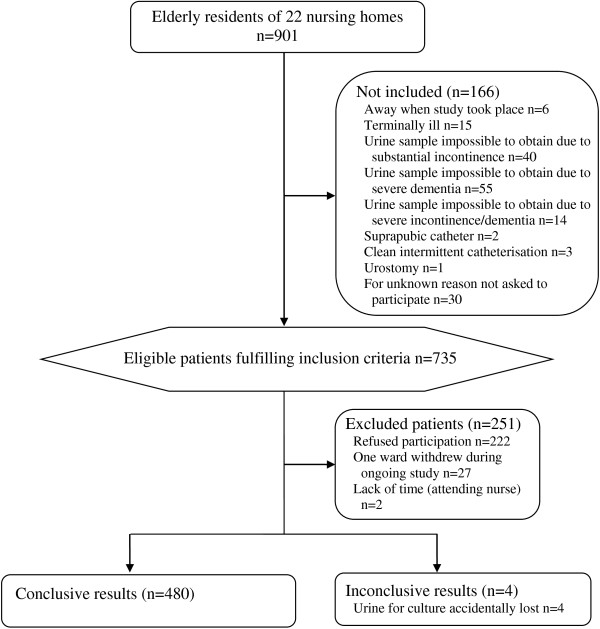
Participant flow chart 2012.

In 2012, 735 of 901 individuals in 22 nursing homes fulfilled the inclusion criteria, with 484 (66%) accepting participation (Figure 2). Urine samples were provided from 480 individuals in 2012 (421 voided urine samples and 59 urine samples from indwelling urinary catheters); 321 (67%) women and 159 (33%) men. Women’s ages (87 years, SD 6.6, range 62–100) were slightly higher than men’s (mean 85 years, SD 7.0, range 65–100) (p = 0.010). 71/480 (15%) suffered from diabetes mellitus, and 253/480 (53%) had dementia. When the urine specimens were collected, 23/480 (4.8%) had ongoing antibiotic treatment. Another 45/480 (9.4%) had no ongoing antibiotic treatment when the urine specimens were collected, but had received antibiotics during the previous month. The presence of new or increased urinary symptoms occurring during the last week was for dysuria 5/480 (1.0%), urinary urgency 6/480 (1.3%), and urinary frequency 2/480 (0.42%).

### Differences between the studied populations in 2003 and 2012

Men’s mean age was 3.1 years higher in 2012 compared to 2003 (p = 0.00016), while there was a statistically insignificant trend towards a higher mean age among women (0.92 year, p = 0.073). The proportion of men was higher in 2012; 33% compared to 26% in 2003 (p = 0.0087).

### Bacterial findings

*E. coli* was by far the most common bacterial species. Bacterial growths in urine cultures and distribution among bacterial species are presented in Tables 1 and 2.

**Table 1:** Bacterial growths in urine cultures from 2003 and 2012

	**Voided urine 2003 N = 651**	**Voided urine 2012 N = 421**	**Indwelling urinary catheter 2012 N = 59**
Negative culture	68% (444)	65% (274)	54% (32)^1^
Sparse growth	2.9% (19)	2.9% (12)	1.7% (1)
Significant growth	29% (188)	32% (135)	44% (26)
Sparse + significant growth	32% (207)	35% (147)	46% (27)

^1^There were mixed growths in all but one of cultures obtained from urinary catheters, classified as negative.

**Table 2:** Bacterial growth in positive urine cultures from voided urine at nursing homes for the elderly

	**========== 2003 ==========Positive cultures**			**========== 2012 ==========Positive cultures**		
	**Sparse (n = 19)**	**Significant (n = 188)**	**Total**^**1**^** (n = 207)**	**Sparse (n = 12)**	**Significant (n = 135)**	**Total**^**1**^** (n = 147)**
*Escherichia coli*	6.8% (14)	62% (129)	69% (143)	5.4% (8)	74% (109)	80% (117)
*Klebsiella* species	0.48% (1)	11% (22)	11% (23)	0	7.5% (11)	7.5% (11)
*Enterococcus faecalis*	1.0% (2)	7.2% (15)	8.2% (17)	0	0.68% (1)	0.68% (1)
*Enterococcus faecium*	0.48% (1)	1.4% (3)	1.9% (4)	0	0	0
*Proteus* species	0	0	0	0	0.68% (1)	0.68% (1)
*Proteus mirabilis*	0	1.4% (3)	1.4% (3)	0	1.4% (2)	1.4% (2)
*Proteus vulgaris*	0	0.48% (1)	0.48% (1)	0	0.68% (1)	0.68% (1)
*Enterobacter* species	0	1.9% (4)	1.9% (4)	0	1.4% (2)	1.4% (2)
*Psuedomonas aeruginosa*	0	1.4% (3)	1.4% (3)	0	0	0
*Citrobacter*	0	0	0	0	1.4% (2)	1.4% (2)
*Serratia liquefaciens*	0	0	0	0	0.68% (1)	0.68% (1)
*Staphylococcus aureus*	0	0	0	0.68% (1)	0.68% (1)	1.4% (2)
Coagulase-negative staphylococci	0	1.9% (4)	1.9% (4)	0	0.68% (1)	0.68% (1)
Alpha-hemolytic streptococci	0	1.0% (2)	1.0% (2)	0	1.4% (2)	1.4% (2)
Group G beta-hemolytic streptococcus	0	0.48% (1)	0.48% (1)	1.4% (2)	0	1.4% (2)
Group C beta-hemolytic streptococcus	0	0	0	0.68% (1)	0	0.68% (1)
Group B beta-hemolytic streptococcus	0.48% (1)	0.48% (1)	1.0% (2)	0	0.68% (1)	0.68% (1)

^1^Growth of bacteria in all positive urine cultures, % (n).

There was no significant difference in percentage of positive urine cultures from voided urine samples in 2003 and 2012; 32% versus 35% (p = 0.29). However, there was a significantly higher proportion of *E. coli* in positive urine cultures from voided urine samples in 2012 compared to 2003; 117/147 (80%) versus 143/207 (69%), p = 0.027.

The proportion of positive urine cultures from voided urine was more common among women than men; 184/482 (38%) versus 23/169 (14%), p < 10^-6^ in 2003 and 134/295 (45%) versus 13/126 (10%), p < 10^-6^ in 2012.

Findings of *E. coli* in positive urine cultures from voided urine were more common among women than men; 134/184 (73%) versus 9/23 (39%), p = 0.00098 in 2003 and 110/134 (82%) versus 7/13 (54%) p = 0.027 in 2012.

### Resistance rates in *E. coli*

The average resistance rates for all tested antibiotics were similar between 2003 and 2012, however there was a non-significant trend (p = 0.090) towards higher resistance rates for cefadroxil in 2012, but still at the low level of 2.6% for all *E. coli* isolates (Table 3). It was not possible to compare resistance rates for quinolones between 2003 and 2012 as nalidixic acid was no longer used as a screening disk for quinolone resistance in 2012.

**Table 3:** **Resistance rates for UTI antibiotics in *Escherichia coli***^**1**^**, voided urine specimens**

	**2003 (n = 143)**^**2**^	**2012 (n = 117)**^**2**^	**P-value**^**3**^
Mecillinam	4.2% (6)	7.7% (9)	0.23
Ampicillin	18% (26)	21% (25)	0.52
Cefadroxil	0.0% (0)	2.6% (3)	0.090
Trimethoprim	13% (18)	12% (14)	0.88
Nitrofurantoin	0.70% (1)	0.85% (1)	1.0
Nalidixic acid	12% (17)	Not tested	---
Ciprofloxacin	Not tested	3.4% (4)	---
Classic ESBL^4^		1.7% (2)	
AmpC^5^		0.85% (1)	

^1^Sparse and significant growth of *Escherichia coli*.

^2^Proportion of resistant *Escherichia coli* % (number).

^3^Pearson chi-square and, when appropriate, Fisher’s exact test.

^4^*E. coli* producing extended spectrum beta-lactamase enzymes.

^5^*E. coli* with plasmid mediated AmpC production.

### ESBL

In 2012, there were two isolates of *E. coli* producing extended spectrum beta-lactamase (classic ESBL), and one isolate with plasmid mediated AmpC production. No carbapenemases were detected. No other ESBL-producing Enterobacteriaceae were found.

In 2003, no ESBL-producing Enterobacteriaceae were found.

### Predictors of UTI antibiotic resistance in *E. coli*

Predictors for antibiotic resistance in voided urine specimens are presented in Table 4.

**Table 4:** **Predictors of UTI antibiotic resistance in *Escherichia coli***^***1 ***^**in voided urine specimens**

	**Unadjusted odds ratios (95% CI; p-value)**	**Adjusted odds ratios (95% CI; p-value)**
**Mecillinam**		
Antibiotics last month^2^	**7.2 (2.4-21; p = 0.00040)**	**7.1 (2.4-21; p = 0.00049)**
Age	1.0 (0.94-1.1; p = 0.65)	1.0 (0.94-1.1; p = 0.62)
Gender^3^	<10^-6^ (0-∞; p = 1.0)	<10^-6^ (0-∞; p = 1.0)
**Ampicillin**		
Antibiotics last month^2^	**5.2 (2.4-11; p = 0.000030)**	**5.2 (2.4-11; p = 0.000036)**
Age	1.0 (0.98-1.1; p = 0.40)	1.0 (0.97-1.1; p = 0.42)
Gender^3^	0.26 (0.033-2.0; p = 0.20)	0.32 (0.040-2.6; p = 0.28)
**Cefadroxil**		
Antibiotics last month^2^	<10^-6^ (0-∞; p = 1.0)	<10^-6^ (0-∞; p = 1.0)
Age	1.1 (0.91-1.4; p = 0.28)	1.1 (0.91-1.4; p = 0.30)
Gender^3^	<10^-6^ (0-∞; p = 1.0)	<10^-6^ (0-∞; p = 1.0)
**Trimethoprim**		
Antibiotics last month^2^	**4.0 (1.7-9.4; p = 0.0018)**	**3.9 (1.6-9.2; p = 0.0023)**
Age	1.0 (0.96-1.1; p = 0.60)	1.0 (0.96-1.1; p = 0.65)
Gender^3^	<10^-6^ (0-∞; p = 1.0)	<10^-6^ (0-∞; p = 1.0)
**Nitrofurantoin**		
Antibiotics last month^2^	1.0×10^8^ (0-∞; p = 1.0)	1.1×10^8^ (0-∞; p = 0.99)
Age	1.2 (0.90-1.6; p = 0.22)	1.3 (0.88-1.8; p = 0.21)
Gender^3^	<10^-6^ (0-∞; p = 1.0)	1.2×10^-5^ (0-∞; p = 1.0)
**Nalidixic acid**^**4 **^**2003**		
Antibiotics last month^2^	**4.3 (1.4-13; p = 0.013)**	**4.6 (1.4-16; p = 0.014)**
Age	**0.92 (0.87-0.98; p = 0.014)**	**0.92 (0.86-0.99; p = 0.018)**
Gender^3^	<10^-6^ (0-∞; p = 1.0)	<10^-6^ (0-∞; p = 1.0)
**Ciprofloxacin**^**5 **^**2012**		
Antibiotics last month^2^	3.1 (0.30-32; p = 0.35)	3.6 (0.32-40; p = 0.31)
Age	1.1 (0.91-1.3; p = 0.38)	1.1 (0.90-1.3; p = 0.36)
Gender^3^	<10^-6^ (0-∞; p = 1.0)	<10^-6^ (0-∞; p = 1.0)

^1^N = 255 (22 sparse growth and 233 significant growth), voided urine specimens in 2003 and 2012 from patients with known antibiotic treatment history.

^2^Any ongoing (n = 8) or previous antibiotic treatment during the last month (n = 25). Reference category: no antibiotics last month.

^3^Reference category: female.

^4^Only includes residents from 2003 since nalidixic acid only was tested in 2003 (n = 138).

^5^Only includes residents from 2012 since ciprofloxacin only was tested in 2012 (n = 117).

Statistically significant findings are bold.

In 2012 any overnight admission to hospital was registered. Cramer’s V between hospital admissions and any antibiotic prescription was calculated, and there was no correlation between “any hospitalisation during the six last months” and “any antibiotic treatment during the six last months”, 0.090 (p = 0.33). Of those in 2012 with *E. coli* in voided urine samples, 23/117 (20%) had been hospitalised during the previous six months. To evaluate the impact of this hospitalisation on antimicrobial resistance rates, logistic regressions were performed, and odds ratios regarding the presence of antibiotic resistance, adjusted for age, gender and any antibiotic treatment during the previous six months, was for ciprofloxacin 13 (1.1-153; p = 0.040), ampicillin 5.2 (1.8-15; p = 0.0021), cefadroxil 8.6 (0.71-103; p = 0.091), trimethoprim 2.4 (0.70-8.5; p = 0.16), mecillinam 2.1(0.48-9.5; p = 0.32), nitrofurantoin 0.0 (0.0-∞; p = 1.0) and any antimicrobial tested for 4.4 (1.6-12; p = 0.0033).

### Resistance rates in *Klebsiella* spp

The resistance rates among isolated *Klebsiella* spp. in voided urine in 2003 were as follows; for ampicillin 96% (22/23), nitrofurantoin 96% (22/23), mecillinam 13% (3/23), nalidixic acid 9.0% (2/23), trimethoprim 0% (0/23) and cefadroxil 0% (0/23).

The resistance rates among isolated *Klebsiella* spp. in voided urine in 2012 were; for ampicillin 91% (10/11), nitrofurantoin 91% (10/11), mecillinam 0% (0/11), ciprofloxacin 0% (0/11), trimethoprim 27% (3/11) and cefadroxil 0% (0/11). Similar rates were seen in 10 *Klebsiella* isolates from indwelling urinary catheters.

### Urine specimens obtained from urinary catheters

In this study 46% (27/59) of the cultures of urine specimens obtained from indwelling urinary catheters were classified as positive. There were growths of mixed bacterial flora in all but one of the cultures obtained from urinary catheters, classified as negative. The bacterial findings in positive urine cultures obtained from urinary catheters were; *E. coli* 48% (13/27), *Klebsiella* spp. 37% (10/27), *Proteus mirabilis* 11% (3/27) and *Enterobacter* spp. 3.7% (1/27).

Resistance rates in *E. coli* (catheter urine specimens) in 2012 were; for ampicillin 46% (6/13), trimethoprim 15% (2/13), mecillinam 0% (0/13), ciprofloxacin 15% (2/13), cefadroxil 7.7% (1/13) and nitrofurantoin 0% (0/13). There was a trend towards higher resistance rates in *E. coli* in urine specimens from catheters compared to voided urine for ampicillin (p = 0.079) and ciprofloxacin (p = 0.11).

## Discussion

### Summary

There were still comparatively low levels of antimicrobial resistance in urinary pathogens among Swedish nursing home residents with no major changes between 2003 and 2012. Any antibiotic treatment during the last month and hospitalisation during the last six months predicted higher resistance rates among urinary pathogens.

### Strengths and limitations of the study

A major strength of this study is that urine samples were collected from all those capable of providing a urine sample at the participating nursing homes for the elderly. Thus, this study describes the native antimicrobial resistance in nursing homes for the elderly. Previously, most studies of antimicrobial resistance in uropathogens among elderly residents of nursing homes compiled antimicrobial resistance in urine cultures taken when the clinician suspected a UTI. Those with UTI symptoms and underlying diseases in the urinary tract comprised a selected patient group assumed to have higher resistance rates.

Another major strength is the possibility to compare resistance rates at nursing homes in the same geographical area between 2003 and 2012. Furthermore, since this study includes many nursing homes they can be assumed to be representative of all Swedish nursing homes, especially whereby nursing homes are similarly organised in all regions of Sweden (municipal care).

In this study we obtained urine specimens and study protocols from 55% (651/1187) of the individuals registered at the nursing homes in 2003, and 53% (480/901) in 2012. This may appear low but is similar to previously published studies in nursing homes for the elderly [31]. One major reason for not participating in this study was substantial urinary incontinence, often combined with dementia. 8.1% (96/1187) and 25% (222/901) of the individuals registered at the nursing homes refused participation in 2003 and 2012, respectively. Still this may be considered acceptable when studying an elderly fragile population with a high proportion of residents with dementia as well as the ethical requirement of approval from appointed representatives/relatives.

### Comparison with existing literature

Several studies have shown increased antimicrobial resistance in uropathogens during the last ten years [2,32]. In this study the average resistance rates for all tested antibiotics were similar between 2003 and 2012. In contrast to most countries the prevalence of ESBL-producing Enterobacteriaceae was low in this study. This may be partly due to successful efforts in Sweden to lower antibiotics usage and the choice of narrow spectrum antibiotics in favour of e.g. ciprofloxacin [23,33].

The overall resistance rates in *E. coli* for UTI antibiotics in Sweden in 2012 [23] as compared to this study (% R in Sweden/% R in this study) were; for ampicillin 31%/21%, mecillinam 4.6%/7.7%, cefadroxil 3.5%/2.6%, nitrofurantoin 1.1%/0.85%, trimethoprim 19%/12% and ciprofloxacin 7.6%/3.4%. Thus, the proportion of resistant bacteria was lower than expected for all antibiotics excepting mecillinam in this study. This might be partly explained by national statistics being based on clinical isolates and our material from all those able to provide a urine specimen. On the other hand a nursing home population can be assumed to have higher antimicrobial resistance rates since they have a high level of co-morbidity and are prescribed more antibiotics than younger individuals. Thus, it is satisfying to note that a Swedish nursing home population still has comparatively low antimicrobial resistance rates among *E. coli*.

Hospitalisation during the previous six months increased the risk for antibiotic resistance in *E. coli* against ampicillin, ciprofloxacin and any antimicrobial tested, adjusted for age, gender and any antibiotic treatment during the previous six months. This is consistent with several articles reporting prior hospitalisation as a risk factor for antimicrobial resistance which suggests that the hospital is a source of antibiotic-resistant organisms [15].

There was a low prevalence of new or increased dysuria and urgency in this study compared to studies collecting urine specimens from patients with suspected UTI. This was expected as it was a screening study collecting urine specimens from all those able to provide a urine sample at the participating nursing homes, regardless of the presence of symptoms.

### Methodological aspects

Procedures utilizing the presence of symptoms or outcomes of prior dipstick testing, to influence the setting of cut-off levels for CFU/mL in urine cultures to label growth as clinically significant, may enhance the diagnostic procedure [34]. This procedure has also been used in previous studies of antimicrobial susceptibility whereby it reflects the clinical situation [6,32,35]. Few individuals had any specific symptom of UTI, but many had significant growth, which is an important result to consider when evaluating urine samples in this population. In this study, when analysing antibiotic susceptibility all isolates with sparse or significant growth were included to identify the pool of resistant bacteria in the nursing home population.

In general, there are some minor differences in breakpoints between 2003 and 2012 due to methodological changes over time. In case of a difference, the breakpoints have only been changed so that a bacterium is more likely to be classified as resistant in 2012 as compared to 2003. For the quinolones, nalidixic acid was no longer used as a screening disk in 2012. Thus, it is not possible to properly compare resistance rates for quinolones during the studied period.

It is inappropriate to compare resistance rates for *Klebsiella* isolates between 2003 and 2012 due to the low numbers of *Klebsiella* spp. in 2012.

*E. coli* was by far the most common finding among all isolated species both in 2003 and 2012. However, the proportion of *E. coli* in voided urine was higher in 2012; 80% (117/147) as compared to 69% (143/207) in 2003. This could in part depend on the lower proportion of *E. faecalis* in 2012; 0.68% (1/147) versus 8.2% (17/207) in 2003. Even if these are small numerical differences, we cannot provide any satisfactory explanation. We have no data indicating more complicating factors within the urinary tract in 2003, since we have not investigated for that.

Urine culture screening for bacteriuria was used in this study to describe native antimicrobial resistance, however, in clinical practice urine specimens should not be considered unless patients have symptoms from the urinary tract.

## Conclusions

There were still comparatively low levels of antimicrobial resistance for urinary pathogens among Swedish nursing home residents with no major changes between 2003 and 2012. Any antibiotic courses during the previous month and hospitalisation during the previous six months predicted higher resistance rates among urinary pathogens in this study. It is important to use antibiotics properly and continue analysing antimicrobial resistance in nursing homes to guide empirical treatment due to the potentially high risk for increasing antibiotic resistance in this population.

## Competing interests

The authors declare that they have no competing interests.

## Authors’ contributions

All authors participated in the design of the study. PDS and ME collected the data. PDS analysed the data and drafted the manuscript. All authors contributed to the interpretation of the analyses, critical reviews and revisions, and final approval of the paper.

## Pre-publication history

The pre-publication history for this paper can be accessed here:

http://www.biomedcentral.com/1471-2318/14/30/prepub
